# SYSTEMS BIOLOGY OF AGING

**DOI:** 10.1093/geroni/igz038.752

**Published:** 2019-11-08

**Authors:** Vyacheslav Labunskyy

**Affiliations:** Boston University School of Medicine, Boston, Massachusetts, United States

## Abstract

The biology of aging is an immensely complex process that impacts function at the sub-cellular, cellular, tissue, organ, and whole organism levels. New technologies and systems-level approaches provide an opportunity to greatly enhance our understanding of this complexity.

